# Middle cranial fossa approach for acoustic neuroma

**DOI:** 10.3171/2021.7.FOCVID21124

**Published:** 2021-10-01

**Authors:** Paul W. Gidley, Joel Z. Passer, Joshua C. Page, Franco DeMonte

**Affiliations:** Departments of 1 Head and Neck Surgery and; 2 Neurosurgery, MD Anderson Cancer Center, Houston, Texas

**Keywords:** acoustic neuroma, middle cranial fossa approach, hearing preservation

## Abstract

The middle fossa approach for the resection of small acoustic neuromas is a viable, but underutilized treatment modality with the goal of hearing preservation. The authors aim to demonstrate this approach and its nuances through this video presentation. A 38-year-old man presented with an incidentally discovered small, intracanalicular acoustic neuroma that was initially observed, but growth was noted. The patient had good hearing, and therefore a hearing preservation approach was offered. A gross-total resection was achieved, and the patient maintained good hearing postoperatively. This video demonstrates relevant anatomy, surgical indications, technical aspects of resection, including reconstruction, and postoperative outcomes.

The video can be found here: https://stream.cadmore.media/r10.3171/2021.7.FOCVID21124

## Transcript

This is Dr. Paul Gidley, and this is our middle cranial fossa approach for excision of acoustic neuroma.

### 0:27 Middle Fossa Anatomy

The middle fossa has a very complex anatomy, which is briefly reviewed here. Internal auditory canal lies at the same level as the external auditory canal. The key landmarks for the middle fossa are labeled here. An imaginary line drawn from the GSPN joins an imaginary line drawn from the arcuate eminence to form an angle that is roughly 90° to 120°. The internal auditory canal is found by bisecting this angle.

### 1:02 Patient Presentation

The patient presented is a 38-year-old man with an incidental acoustic neuroma first identified in 2012. It was observed to grow. This is his initial MRI showing a small intracanalicular tumor. Repeat imaging shows interval growth of the tumor. The audiogram shows good hearing in the right ear with a word understanding of 96%. ABR shows absent wave III and wave V on the right side.

### 1:43 Management Options

The options for management were explained to the patient, and these include observation with serial imaging, microsurgical resection, and stereotactic radiosurgery. The risk and benefits of each one of these approaches was outlined, and he opted for surgical excision.

### 2:03 Hearing Preservation Indications

Hearing preservation is offered to patients under the age of 65 with small tumors and good hearing. ABR waveforms should be intact to allow intraoperative monitoring.

### 2:19 Patient Preparation

The patient is positioned supine with the head in a Mayfield head holder. Electrodes for facial nerve monitoring are placed. ABR is performed intraoperatively. In this patient, the intraoperative waves I, III, and V were present. The skin is marked to outline a posteriorly based skin flap that follows the hairline.

### 2:42 Incision/Craniotomy

Temporalis fascia is harvested and set aside. An anterior temporalis muscle flap is elevated. Craniotomy is planned to be centered at the external auditory canal measuring 4 cm high and 4 cm wide. It is important that the sides of the craniotomy are parallel.

### 3:08 Dural Elevation/Identification of Middle Fossa Anatomy

The patient is then given mannitol and is hyperventilated to allow dura elevation. Dura elevation is performed from a posterior-to-anterior direction. Dura is elevated until the posterior fossa, arcuate eminence, and middle meningeal artery are identified. A House-Urban retractor is then placed at the petrous apex. The middle fossa anatomy can then be examined. The arcuate eminence and the GSPN are marked here. Imaginary lines outlining these two structures are marked here, with bisection here, which should overlie the IAC.

### 3:57 Drilling

Drilling starts over the arcuate eminence in order to blue-line the superior semicircular canal. The superior semicircular canal is always perpendicular to the posterior petrous ridge. Any air cells that are opened during this dissection are sealed off with bone wax. The blue line of the canal is seen here. Drilling then continues anterior and medial to the superior semicircular canal to open up the air cells of the petrous apex. This drilling continues until the dura of the posterior fossa is encountered. The bone around the porus is completely removed, exposing the dura. Drilling is then continued laterally along the internal auditory canal. Drilling continues until Bill’s bar is reached. The remaining thin shell of bone over the internal auditory canal is removed with Fisch instruments.

### 5:17 Dural Opening/Tumor Resection

The dura of the internal auditory canal is then opened with a 5910 Beaver blade. The dura is reflected to show the facial nerve anteriorly and superior vestibular nerve posteriorly. A Prass probe is used to identify the facial nerve and to separate it from the superior vestibular nerve. The superior vestibular nerve is reflected posteriorly, and the tumor can be seen between it and the facial nerve. The superior vestibular nerve is then cut with microscissors to allow better visualization of the tumor from the inferior vestibular nerve. The tumor can then be elevated out of the internal auditory canal by severing its lateral attachment to the inferior vestibular nerve. The tumor is then carefully and bluntly separated from the facial nerve and the cochlear nerve, which is seen here as a bright white, densely myelinated nerve. The facial nerve is then stimulated with a Prass probe to ensure its integrity.

### 6:44 Reconstruction

A portion of temporalis muscle is then harvested. This muscle is used to plug the defect in the internal auditory canal. Temporalis fascia is laid over the muscle plug to reconstruct the middle fossa. A bone chip is then placed over the defect in the petrous apex and the middle fossa retractor is then removed. The craniotomy flap is placed back into its anatomical location, rigidly fixated with plates. The temporalis muscle and skin flaps are then closed in a watertight fashion.

### 7:36 Postoperative Management

Patients are observed overnight in the ICU. An MRI is obtained the following day and the patient is observed another 3 or 4 days until they meet discharge criteria.

MRI 3 years later shows no evidence of disease. Postoperative audiogram shows class B hearing, with an SRT of 35 and a discrim score of 88%.

In our practice, we have found that patients with class A or B hearing preoperatively are able to maintain class A or B hearing in 67% of cases after undergoing middle cranial fossa resection of acoustic neuroma, consistent with other modern reports.

### 8:13 References^1–10^

## Disclosures

The authors report no conflict of interest concerning the materials or methods used in this study or the findings specified in this publication.

## Author Contributions

Primary surgeon: Gidley, DeMonte. Editing and drafting the video and abstract: Gidley, Passer, Page. Critically revising the work: all authors. Reviewed submitted version of the work: all authors. Approved the final version of the work on behalf of all authors: Gidley.

## References

[1] Committee on Hearing and Equilibrium guidelines for the evaluation of hearing preservation in acoustic neuroma (vestibular schwannoma) American Academy of Otolaryngology–Head and Neck Surgery Foundation, Inc. Otolaryngol Head Neck Surg 1995 113 3 179 180 7675475 10.1016/S0194-5998(95)70101-X

[2] DeMonte F Gidley PW Hearing preservation surgery for vestibular schwannoma: experience with the middle fossa approach Neurosurg Focus 2012 33 3 E10 10.3171/2012.7.FOCUS12172 22937844

[3] Dowling EM Patel NS Lohse CM Durability of hearing preservation following microsurgical resection of vestibular schwannoma Otol Neurotol 2019 40 10 1363 1372 31725593 10.1097/MAO.0000000000002378

[4] Gantz BJ Parnes LS Harker LA McCabe BF Middle cranial fossa acoustic neuroma excision: results and complications Ann Otol Rhinol Laryngol 1986 95 5 Pt 1 454 459 3767216 10.1177/000348948609500504

[5] House WF Surgical exposure of the internal auditory canal and its contents through the middle, cranial fossa Laryngoscope 1961 71 1363 1385 14036379 10.1288/00005537-196111000-00004

[6] House WF Gardner G Hughes RL Middle cranial fossa approach to acoustic tumor surgery Arch Otolaryngol 1968 88 6 631 641 5724837 10.1001/archotol.1968.00770010633011

[7] Kutz JW Jr Scoresby T Isaacson B Hearing preservation using the middle fossa approach for the treatment of vestibular schwannoma Neurosurgery 2012 70 2 334 341 21826031 10.1227/NEU.0b013e31823110f1

[8] Meyer TA Canty PA Wilkinson EP Hansen MR Rubinstein JT Gantz BJ Small acoustic neuromas: surgical outcomes versus observation or radiation Otol Neurotol 2006 27 3 380 392 16639278 10.1097/00129492-200604000-00015

[9] Raheja A Bowers CA MacDonald JD Middle fossa approach for vestibular schwannoma: good hearing and facial nerve outcomes with low morbidity World Neurosurg 2016 92 37 46 27150655 10.1016/j.wneu.2016.04.085

[10] Roche JP Woodson EA Hansen MR Gantz BJ Ultra long-term audiometric outcomes in the treatment of vestibular schwannoma with the middle cranial fossa approach Otol Neurotol 2018 39 2 e151 e157 29315191 10.1097/MAO.0000000000001678

